# Basic microsurgical skills can be taught to novices with video material only - a prospective multicenter laboratory study

**DOI:** 10.1016/j.bas.2025.105910

**Published:** 2025-12-16

**Authors:** Andrei Schildt, Anni Pohjola, Ville Vasankari, Jenni Määttä, Anna Seidlová, Martin Májovský, Norbert Svoboda, David Netuka, Ahmad Hafez, Martin Lehecka

**Affiliations:** a Department of Neurosurgery, Helsinki University Hospital, P.O. Box 266, FI-00029, Helsinki, Finland; b Department of Neurosurgery and Neurooncology, First Faculty of Medicine, Charles University and Military University Hospital, Prague, Czech Republic

**Keywords:** Video education, Neurosurgery, Surgical training, Exoscope

## Abstract

**Introduction:**

The increasing use of educational video materials has provided an alternative to traditional apprenticeship-models in surgical education. We aim to evaluate if video materials are as effective as hands-on tutoring in teaching basic microsuturing skills. Additionally, we want to see if the magnification device (microscope vs. exoscope) affects learning outcomes.

**Research question:**

Is video-based instruction an effective alternative to hands-on tutoring in teaching basic microsuturing skills to novices?

**Materials and methods:**

We designed an 8-h training program with videos to teach basic microsuturing skills to novices. Thirty medical students from two large medical universities in Europe (Helsinki and Prague) were randomised to receive either video instructions only (n = 20) or hands-on tutoring and video instruction (n = 10). Participants were further assigned to using either an exoscope (n = 15) or a microscope (n = 15) in their training. We assessed skill acquisition using a standardized microsuturing test task. All tasks were recorded and scored based task speed, quality of the suturing and the number of errors.

**Results:**

All groups demonstrated significant improvement in suturing speed, qualitative assessment, and fewer errors. Video instructions only produced non-inferior results to hands-on tutoring in the improvement in speed, error count and quality of sutures. No significant differences were found when comparing microscope and exoscope users.

**Discussion and conclusion:**

Video-based instruction and hands-on tutoring seem equally effective in teaching basic microsuturing to surgical novices, irrespective of the magnification device used. Video-materials can be utilized in microsurgical laboratory training of novices as a more resource-efficient teaching method compared to hands-on tutoring.

## Highlights

• Video-based instruction of microsuturing is non-inferior compared to hands-on tutoring.
• A short video-instruction program resulted in significant improvement in microsuturing.
• Introduction of 3D exoscopes to microsurgical training does not hinder skill development.

## 1 Introduction

Traditionally, surgical training has followed an apprenticeship model in which students progress from observation to active participation (Evans and Schenarts, 2016; Walter, 2006; Youssef et al., 2023). This method of training requires the presence of an experienced instructor, making it more resource-demanding and difficult to scale to a larger audience. In recent years, education technology has seen a rise in the use of remote teaching methods, such as video materials, along with the COVID-19 pandemic (Koh and Daniel, 2022). These remote methods are potentially a more resource-efficient method of teaching. While high-quality video materials have been found to be effective in the teaching of laparoscopic and open surgical skills (Youssef et al., 2023; Ahmet et al., 2018; Akl et al., 2008; Alameddine et al., 2018; Chien et al., 2015; Crawshaw et al., 2016; Dantas et al., 2010; Hamour et al., 2018; Nousiainen et al., 2008; van Det et al., 2011), their role in microsurgical education remains unclear.

The emergence of 3D exoscopes has expanded the microsurgical training landscape, raising questions about the optimal timing for their integration into practice. Compared to the traditional operating microscope, it has been reported that the 3D exoscope provides superior ergonomics and visualization of the surgical field (Moisi et al., 2017), while producing comparable outcomes in various neurosurgical pathologies (Calvanese et al., 2024a, 2024b; Haeren et al., 2022; Muhammad et al., 2019; Rossmann et al., 2023; Vasankari et al., 2025; Veldeman et al., 2024). Prior research has not evaluated if training using the exoscope produces similar results in novices as the traditional operating microscope. If the learning curve of the exoscope can be proven comparable, future surgical residents could benefit from its early adoption into training.

In this prospective, international, multicenter laboratory study, our aim is to evaluate the effectiveness of high-quality instructional in teaching basic microsurgical skills to novices. In addition, we want to compare the skill development between the participants training with a traditional operating microscope and the digital 3D exoscope. We hypothesize that (i) a short, structured training course will enable rapid skill acquisition among novices, (ii) video-based instructions will be non-inferior to hands-on tutoring, and (iii) performance will be similar between exoscope and microscope users.

## 2 Materials and methods

### 2.1 Participants

We recruited 30 medical students from the University of Helsinki (n = 20; 1st-3rd-year) and from Charles University (n = 10; 4th-6th). Exclusion criteria were previous experience in surgery and use of the exoscope or operating microscope. We adhered to the Declaration of Helsinki. All participants gave their informed consent to participation, and no data allowing identification of the participants was collected.

### 2.2 Study design

The participants were divided into three groups (10 participants/group) to receive either on-site microsurgical tutoring by a mentor in addition to video instruction (Helsinki n = 10) or video instruction only (Prague n = 10, Helsinki n = 10). The allocation of a smaller sample to the on-site tutoring group was due to resource-based restraints. Furthermore, we divided the participants evenly into those using the exoscope (Helsinki: Aesculap AEOS®, B. Braun, Melsungen, Germany; Prague: ORBEYE™, Olympus, Tokyo, Japan) and the microscope (Leica M320 surgical microscope, Leica Microsystems GmbH, Wetzlar, Germany) for the duration of their training. The final groups are seen in Table 1.

**Table 1:** Distribution of participants into different training groups.

Training Modality	Microscope	Exoscope
Hands-on tutoring + video	N = 5	N = 5
Video instructions only	N = 10	N = 10

### 2.3 Training program and video material

The training program focused on adapting to the use of a magnification device, microsurgical instruments, and the basic skill of microsuturing. The program consisted of four training sessions (2h/session), making the total length of the program 8 h. Consecutive training sessions took place at a maximum of two weeks apart, and the program was completed within a month. Each training session was accompanied by learning objectives and specific exercise tasks. The tasks were getting gradually more difficult as the course progressed. All groups followed the same structured course (Table 2).

**Table 2:** Contents of each training session.

Training session	Exercise	Learning objectives	Duration of corresponding video
1	Suturing of a longitudinal slit on a silicone glove using 6-0 or 7-0 size thread and a magnification of 8-16x.	To become comfortable with microinstruments and be able to place simple interrupted micro sutures.	6:35
2	Suturing of a longitudinal slit on a silicone glove using 7-0 or 8-0 size suture and a magnification of 16-25x.	To perform a simple suture using increased magnification and begin to pay attention to the quality criteria of a good suture.	2:01
3	Suturing of a slit on a silicone tube using 8-0 or 9-0 size suture and a magnification of 16-25x.	To suture a silicone tube without damaging its lumen. Focus is also on the minimization of unnecessary movements and the use of higher magnification.	3:16
4	Suturing of a circular cutout onto a silicone glove using 8-0 or 9-0 size suture and a magnification of 16-25x.	To apply the skill of tying a simple interrupted suture to more advanced tasks, and increased comfort with the test task.	5:48

We produced a total of four short (<7 min/video, total 17min 40s) educational videos, one for each training session. Both video and tutor groups had access to the video materials, whereas only the tutor group received hands-on instruction during the practice sessions. The participants were instructed to watch the specific video before the corresponding training session and were also encouraged to actively use the videos during the training. Each video contains a demonstration of a new task by a senior neurosurgeon (A.H.), along with content related to the learning objectives. The videos are included in the supplementary materials (Supplementary Videos 1–4).

Supplementary data related to this article can be found online at https://doi.org/10.1016/j.bas.2025.105910

The following are the Supplementary data related to this article:

Multimedia component 2 Multimedia component 2

Multimedia component 3 Multimedia component 3

Multimedia component 4 Multimedia component 4

Multimedia component 5 Multimedia component 5

The tutor group received instruction and guidance throughout each training session from two senior neurosurgical residents (A.P., V.V.) and one consultant neurosurgeon (J.M.).

Participants used their assigned magnification device (exoscope or microscope) in all training sessions and test tasks. No crossover between the devices was allowed, and all training was limited to the planned 2-h sessions without additional practice opportunities.

### 2.4 Test task

We used a specially designed test task to evaluate progression of the participants. Each participant performed the same test task twice, at baseline and following the completion of the training program.

**Fig. 1:**
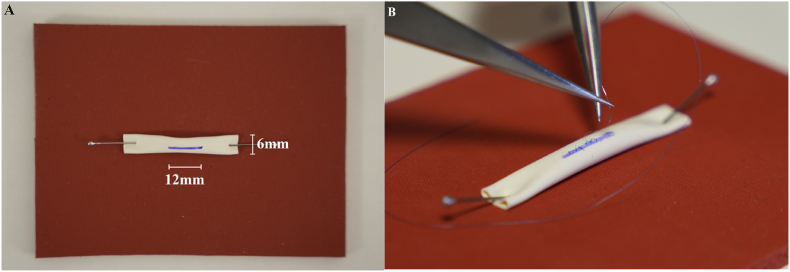
(A) Model used for test task, the diameter of the tube was 6 mm and a line (12 mm) was drawn along which participants could cut the slit. (B) The silicone tube was attached to a flat surface using pin needles.

**Fig. 2:**
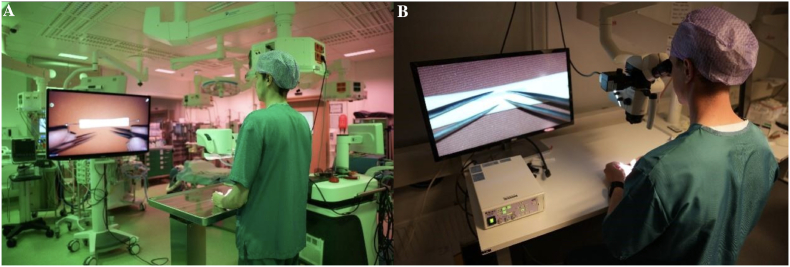
(A) Exoscope set up used for test task completion. (B) Microscope set up used for test task completion.

The test task consisted of placing 12 simple interrupted sutures on a 12 mm slit in a 6 mm silicone tube, using 8-0 monofilament thread. There was no restriction to the magnification used in the test task. Each participant used two identical jeweler's forceps and microscissors. Participants were also informed of the grading criteria before executing the test. A picture of the model and set up for the task can be seen in Fig. 1A and B. The set up used for the exoscope-assisted task is seen in Fig. 2A, while the microscope-assisted task is seen in Fig. 2B. Video instructions for the test task are also found in the supplementary materials (Supplementary Video 5).

Supplementary data related to this article can be found online at https://doi.org/10.1016/j.bas.2025.105910

The following are the Supplementary data related to this article:

Multimedia component 6 Multimedia component 6

Prior to completing the pre-course test task, participants were allowed to practice by doing three simple interrupted 6-0 sutures and three 8-0 sutures on a silicone glove. Similarly, before completing the post-course test, the participants were also allowed to practice by doing six 8-0 sutures. All participants were given access to the educational videos prior to the test task. The test tasks were completed on separate occasions from the training sessions and there was a maximum of 12 days between the training sessions and the test tasks. All performances were video recorded.

### 2.5 Video analysis

All recorded test performances were evaluated by a single researcher (A.S.) using predefined parameters listed in Table 3. These parameters assessed speed, number of errors, a separate qualitative assessment grade and the quality of the sutures. To calculate the mean absolute deviation (MAD), images of the completed test tasks were obtained with an in-frame measurement scale. Because of the microscopic scale of the measured distances, values were first recorded in pixels using image analysis software and then converted to millimetres. The number of errors was also calculated, with error types being predefined as needle drops, losses of thread, unnecessary penetrations of the tube and instances of moving both instruments out of the field of view. In addition to these quantitative measures, a qualitative evaluation of procedural smoothness, instrument handling, knot tying, and tissue handling was performed. Each subsection of the qualitative assessment was given a grade from one to three, with one representing complete lack of proficiency in the area and three representing a flawless performance.

**Table 3:** Parameters used.

Parameter	Definition
Total time	Time from the first needle puncture to the last thread cut.
Quality: MAD of Bite Size	Mean absolute deviation of the distance between the entry and exit points for all 12 sutures.
Quality: MAD of Suture Spacings	Mean absolute deviation of the distance between two consecutive points of entry.
Number of Errors: Unnecessary Penetration of Tube	The number of times the tube was penetrated unnecessarily.
Number of Errors: Needle Drops	No. of times the needle was unintentionally lost or dropped.
Number of Errors: Losing the Thread	No. of times the thread was unintentionally lost or dropped.
Number of Errors: Both Instruments Out of View	No. of times both instruments left the field of vision.
Qualitative Assessment: Smoothness	A subjective score from 1 to 3 assessing the overall smoothness of the performance.
Qualitative Assessment: Tissue Handling	A subjective score from 1 to 3 assessing the exhibited respect for tissue, treating the tube as a real vessel.
Qualitative Assessment: Instrument Handling	A subjective score from 1 to 3 assessing the correctness of the handling of instruments.
Qualitative Assessment: Knot	A subjective score from 1 to 3 assessing the fluency of knot tying.
Qualitative Assessment: Total	Sum of all other qualitative assessment scores.

### 2.6 Statistical analysis

Continuous variables were tested for normality using the Shapiro-Wilk test for normality. Levene's test was used for equality of variances. Means and standard deviations (SD) are reported for normally distributed parameters, medians and interquartile ranges (IQR) for non-normal parameters.

Baseline and post-training results were compared using paired *t*-test (normally distributed parameters) and Wilcoxon test for non-normal parameters. Analysis groups were based on teaching modality and/or magnification device.

Two-way ANOVA was performed to test for differences (baseline minus post-training results) for parameters meeting assumptions (normality and equivalence of variances) by teaching modality and magnification device. Kruskal-Wallis was used to test differences between groups for non-normally distributed parameters.

A multiple-testing strategy was defined a priori, with an overall significance level of α = 0.05. Bonferroni correction was applied to Kruskal-Wallis test to control for multiple comparisons. For the two-way ANOVA, which examined main effects and interactions for each parameter separately, no additional correction was applied beyond the model's inherent structure, as it was the primary method for testing hypothesis (iii); significant effects would have been followed by post-hoc Tukey's HSD tests with family-wise error control. Within-group (pre–post) improvements were assessed with paired t-tests or Wilcoxon signed-rank tests for each parameter separately. Because these tests directly addressed the primary hypotheses (i) and (ii) and were limited in number, no formal correction was applied.

Statistical analysis was conducted using R (Version 4.4.0) (R Core Team, 2024) in RStudio (Version, 2024.12.0 + 467) (Posit team, 2024). R packages used were tidyverse (v2.0.0), ggplot2 (v3.5.2), car (v3.1-03) and effectsize (v.1.0.1).

## 3 Results

### 3.1 Baseline performance

At baseline the test performances were comparable in both groups (tutor n = 10, video-based n = 20), there were no significant differences in task duration, MAD of suture spacings, MAD of bite sizes, total errors or qualitative assessment scores. Likewise, when grouped by magnification device (exoscope n = 15, microscope n = 15), there were no differences between baseline performances in any parameter. A summary of the baseline results can be seen in Table 4.

**Table 4:** Baseline results.

Parameter	Tutor (n = 10)	Video (n = 20)	P-value	Exoscope (n = 15)	Microscope (n = 15)	P-value
Test Task Time (min)	58.9 [49.0–72.4]	52.8 [40.4–67.4]	0.416	56.4 [41.7–62.0]	60.8 [39.8–78.1]	0.709
Total Number of Errors (n)	96.5 [71–141]	72 [48.5–81.5]	0.055	77 [58–120]	71 [46–89]	0.237
MAD^a^ of Bite Size (mm)	0.21 (SD 0.10)	0.25 (SD 0.15)	0.460	0.28 (SD 0.13)	0.20 (SD 0.13)	0.106
MAD^a^ of Suture Spacings (mm)	0.16 [0.14–0.18]	0.27 [0.13–0.45]	0.302	0.19 [0.13–0.36]	0.17 [0.14–0.33]	0.879
Qualitative Assessment Total	4 [4–4]	4 [4–5]	0.096	4 [4–4]	5 [4–5]	0.030

a MAD = mean absolute deviation.

### 3.2 Improvement after the training program

#### 3.2.1 Video-instruction vs. tutoring

**Fig. 3:**
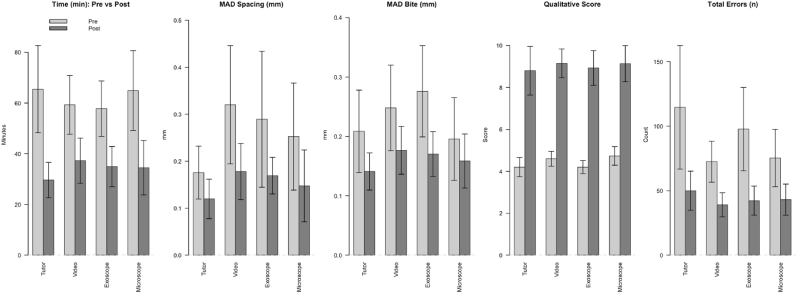
Baseline vs. post-training test results by magnification device (exoscope or microscope) and by teaching modality (tutor or video-based).

Task completion times improved significantly across both video and tutor groups (Fig. 3). In the tutor group, completion times decreased from 59 min [IQR: 49–72] to 28 min [IQR: 22–36] (p < 0.001). In the video group, completion times decreased from 53 min [IQR: 40–67] to 35 min [IQR: 22–48] (p < 0.001). Errors were reduced by 56 % (p = 0.006) in the tutor group, and by 46 % (p < 0.001) in the video group. Qualitative assessment scores also improved significantly in both groups (both p < 0.001).

For MAD of bite size, improvements were not significant in either tutor (p = 0.103) or video group (p = 0.058). For MAD of suture spacing, the video group improved significantly (p = 0.021), whereas the tutor group did not (p = 0.113). Improvements in the different parameters are summarized in Table 5.

**Table 5:** Summary of results grouped by magnification device and teaching modality. Participants are divided by magnification device (exoscope vs. microscope, regardless of teaching modality), and by teaching modality (tutor vs. video, regardless of magnification device).

Hands-on Tutoring vs. Video Learning		
Parameter	Tutoring (n = 10)	Video (n = 20)
Δ Test task time (min)	−28.9 [–26.2 to −41.3]	−21.7 [–12.4 to −29.8]
Δ Total Number of Errors (n)	−63.5 [-37 to −78]	−31.5 [-11 to −50]
Δ MAD^a^ of Bite Size (mm)	−0.07 (SD 0.12)	−0.07 (SD 0.15)
Δ MAD^a^ of Suture Spacings (mm)	−0.07 [0.02 to −0.11]	−0.05 [0.02 to −0.29]
Δ Qualitative Assessment Total	4.60 (SD 1.58)	4.55 (SD 1.32)

**Exoscope vs. Microscope**		

**Parameter**	**Exoscope (n=15)**	**Microscope (n=15)**

Δ Test task time (min)	−26.5 [-14.1 to −30.1]	−23.2 [-19.8 to −41.3]
Δ Total Number of Errors (n)	−50 [-30–72]	−24 [-11 to −50]
Δ MAD^a^ of Bite Size (mm)	−0.11 (SD 0.15)	−0.04 (SD 0.13)
Δ MAD^a^ of Suture Spacings (mm)	−0.05 [0.03 to −0.22]	−0.07 [0.02 to −0.17]
Δ Qualitative Assessment Total	4.73 (SD 1.44)	4.40 (SD 1.35)

a MAD = mean absolute deviation.

#### 3.2.2 Exoscope vs. microscope

When grouped by magnification device, we observed similar improvements (Fig. 3). Task completion times decreased from 56 min [IQR: 42–62] to 31 min [IQR: 24–43] in the exoscope group (p < 0.001), and from 61 min [IQR: 40–78] to 32 min [IQR: 20–43] in the microscope group (p < 0.001). Error counts decreased by 46 % (p = 0.001) in the exoscope group, and by 43 %, (p = 0.004) in the microscope group. Qualitative assessment scores also increased in both exoscope and microscope groups (both p < 0.001).

For MAD of bite size, a significant reduction was observed in the exoscope group (p = 0.018), but not in the microscope group (p = 0.292). For MAD of suture spacing, no significant improvements were detected (exoscope p = 0.069; microscope p = 0.083).

### 3.3 Between-group differences

No significant main effects were found in two-way ANOVA, indicating that neither teaching modality nor magnification device independently impacted the parameters. No interactions were found, meaning that the effect of teaching modality was not dependent on magnification device (all p ≥ 0.125). Detailed ANOVA results are seen in Supplementary Table 1.

For variables not meeting ANOVA assumptions (Δ time, Δ errors, Δ MAD of spacing), subgroup analyses were conducted across the four combinations of teaching modality and magnification device. These analyses did not yield any significant differences.

## 4 Discussion

Our study demonstrated improvement in basic microsuturing skills of surgical novices after a short, dedicated training course. Video-based instructions alone were equally effective as hands-on tutoring and magnification device (microscope vs. exoscope) did not affect the training outcome.

### 4.1 Video-based instruction vs. hands-on tutoring

Video-based instruction has been found beneficial in surgical education, mainly in “macro” surgery. In two different randomized controlled trials on macroscopic wound suturing by medical students, the use of video-based guidance led to similar results compared to live workshops or expert instructions (Chien et al., 2015; Nousiainen et al., 2008). For microsuturing, there is only one randomized trial comparing video-based training program to a tutor-supervised 4-day course among 77 medical students (Dąbrowski et al., 2022). The results were similar to ours, with video instructions resulting in non-inferior results compared to the hands-on tutoring. Compared to our study, there was no comparison of different magnification devices, the video-materials (3h 10 min vs. 18 min) and training sessions (4 days vs. 8 h) were significantly longer, and there were no remote teaching sites.

Video-based instructions have several advantages over classical tutor-based teaching. In addition to demonstrating ideal executions in specific surgical tasks, educational videos can be replayed, paused and closely analyzed for the troubling segments. They can be easily distributed and scaled to larger groups unlike the labor heavy hands-on tutoring, which makes them a viable option even for resource-restricted environments where instructors may not be available. They can be also used remotely as demonstrated by our work. For more advanced training and complex tasks, hands-on supervision will still remain crucial. The instructor's role becomes increasingly important in later stages of training, as it remains the only way for the trainees to receive immediate feedback on their technique. Lastly, there remains a discrepancy between the laboratory environment and the operating room, and improvements in the former may not directly translate to the latter.

### 4.2 Exoscope vs. microscope

The magnification device did not affect the learning of basic microsurgical skills. It has been previously suggested that operating with an exoscope demands more hand-eye coordination than the surgical microscope (Rösler et al., 2022). We did not observe such a trend in our work. It may be related to age and previous experience of the test subjects. The younger “digital native” generation of trainees may be more comfortable working with screens, as reported by a recent study where novices with experience in video-gaming adapted faster to the use of exoscopes (Yousfi et al., 2025a). We believe that introducing exoscopes in the early steps of microsurgical training is beneficial.

Previous studies have evaluated the adaptation to the 3D-exoscopes (Yousfi et al., 2025a; Vasankari et al., 2024; Silva et al., 2023). In a study on the effect of long-term training with the exoscope on a microsurgical dissection task, significant improvement in speed and quality parameters were found over a 12-month period (Silva et al., 2023). In a study on experimental exoscope-assisted end-to-side anastomosis among microsurgeons with different levels of experience, significant improvement was observed after only 6 h of practice, especially among the novices (Vasankari et al., 2024). In the clinical series, the exoscope has been found to be non-inferior to the microscopes in various neurosurgical procedures (Calvanese et al., 2024a, 2024b; Haeren et al., 2022; Muhammad et al., 2019; Rossmann et al., 2023; Vasankari et al., 2025; Veldeman et al., 2024). These findings align with our data, suggesting that even short-term training with the exoscope leads to fast adaptation.

### 4.3 Microsurgical skill acquisition in novices

Basic microsurgical skills can be achieved in novices with short but regular training sessions. Previous studies have found longer training courses (e.g. lasting days or weeks) efficient in teaching microsurgical skills for beginners (Dąbrowski et al., 2022; Yousfi et al., 2025b). Our results support these findings, suggesting that even an 8-h structured training course may be enough to learn the basics of microsuturing. More advanced skills will naturally require way more time and repetitions. In more complex neurosurgical tasks, video materials could be utilized more widely to demonstrate ideal microsurgical techniques and error-avoidance strategies in a standardized manner. Video materials utilizing narrated procedures and stepwise breakdowns of real surgical cases may also support learners in preparing for real-life scenarios by allowing them to repeatedly observe rare or technically demanding steps that are not easily recreated in short courses.

### 4.4 Limitations

We acknowledge some limitations. First, the relatively small study population increased the influence of individual outliers and limited statistical analysis, increasing the risk of type II error. Additionally, the uneven split into tutor and video only groups was based on feasibility and practical limitations associated with hands-on tutoring. The design prioritized gathering data on the scalable video intervention while also allowing for the secondary investigation of the magnification devices. While this group size split may have affected the statistical power, the demonstrated significant improvements in both groups and the absence of significant between-group differences in the two-way ANOVA suggest the training modality and magnification device did not independently impact the outcome. Second, the participants in Prague did not receive a formal warm-up before beginning baseline and post-training test tasks, which may have affected their performance and contributed to an exaggerated appearance of improvement. However, all participants in Prague were subject to the same conditions, allowing for valid within-group comparisons. Third, grouping two different exoscope systems (Olympus ORBEYE and Aesculap AEOS) into a single category represents a methodological simplification, as the devices differ in their intraoperative positioning, ergonomics, and user interfaces. Fourth, because the magnification device was clearly identifiable in the video recordings, blinding the assessments to device type was not possible. Lastly, the instructional videos did not demonstrate the principles of exoscope use and posture in the same manner as for microscope, which may have placed the exoscope group at a relative disadvantage and limited their learning.

## 5 Conclusion

Video-based instruction only and hands-on tutoring seem to be equally effective in teaching basic microsuturing to surgical novices irrespective of the magnification device used. Introduction of 3D exoscopes to microsurgical training does not hinder skill development. Video-materials should be utilized more in the microsurgical laboratory training of novices even as remote teaching.

## Funding

The work was supported by the Neurocenter of Helsinki University Hospital (grant number Y223525029).

## Declaration of competing interest

The authors declare the following financial interests/personal relationships which may be considered as potential competing interests: Andrei Schildt reports financial support was provided by Helsinki University Hospital Neurocenter. If there are other authors, they declare that they have no known competing financial interests or personal relationships that could have appeared to influence the work reported in this paper.
