# Supplementary material for: Basic microsurgical skills can be taught to novices with video material only - a prospective multicenter laboratory study
Source: Brain Spine. 2025 Dec 16;6:105910. doi: 10.1016/j.bas.2025.105910 (PMC12775985; doi:10.1016/j.bas.2025.105910)
Supplement: Multimedia component 1 [file mmc1.docx]

Supplementary Table 1: Two-way ANOVA was performed for parameters meeting assumptions of normality and homogeneity (Δ MAD of bite size and Δ qualitative assessment total). Two-Way ANOVA summary results for each performance metric. Effect sizes were small (partial η² ≤ 0.088). Difference in total time and total errors did not meet the normality assumption of ANOVA and were therefore excluded.

| **Metric** | **Teaching Modality p** | **Group η²** | **Magnification Device p** | **Device η²** | **Interaction p** | **Interaction η²** |
| --- | --- | --- | --- | --- | --- | --- |
| Difference in MAD* of Bite Size | 0.947 | 0.0002 | 0.202 | 0.0643 | 0.376 | 0.0315 |
| Difference in Sum of Qualitative Scores | 0.926 | 0.0003 | 0.515 | 0.0165 | 0.125 | 0.0884 |
| *MAD = mean absolute deviation | | | | | | |
